# Current News and Opinion

**Published:** 1882-02

**Authors:** 


### CURRENT NEWS AND OPINION.

DR. P. BRIQUET died recently in Paris at the age of eighty-six.

DR. ALFRED MCCLINTOCK, of Dublin, Ireland, died on October 21, 1881.

DR. JAMES M. SHEARER died in Dillsburg, Pa., on December 14th, 1881.

M. FEREOL recently reported a case of syphilis inoculated by skin-grafting.

DR. W. N. AMES reports a case of croup successfully treated with jaborandi.

DR. EDWARD REYNOLDS died in Boston, Mass., on Christmas day, aged 88 years.

DR. THOMAS W. GRIMES died at Columbus, Ga., December 12, 1881, aged 73 years.

THEY MULTIPLY.—M. Weiss has discovered a specific *microbe* in the pus of gonorrhœa.

DR. WOOLSEY JOHNSON has been appointed one of the Health Commissioners of New York.

AN EFFORT is being made to secure the appointment of Dental Surgeons to the Army and Navy.

DR. DANIEL HARWOOD, died at Dorchester, Mass., October 2, 1881, in the eighty-first year of his age.

DR. I. I. HAYES, the distinguished arctic explorer, died suddenly in New York, on the 17th of December.

SMALL-POX is increasing in Baltimore and Richmond, Va. It is quite prevalent throughout the country.

THE COUNTRY PRACTITIONER has been discontinued on account of failing eyesight on the part of the editor, Dr. E. P. Townsend.

FOUR TESTICLES enclosed in two perfectly normal scrota were recently found in a patient in the San Fernando Hospital in Sevilla.

SOLINKOFF (*Lancet*, December 3, 1881) has proven experimentally that urea "enters the circulation with the blood which issues from the liver."

A ST. LOUIS Mrs. Malaprop says she likes homeopathic physicians for infantry, but she prefers an allopath when it comes to adultery.

IN OHIO, RECENTLY, a judge refused to compel a medical witness to give expert testimony unless the witness received the fee of an expert.

THE MEDICAL NEWS, the successor of the *Medical News and Abstract* has made its appearance. It presents abundant signs of active life.

MR. CHARLES ABBEY, the senior member of the firm of Charles Abbey & Son, gold foil manufacturers, died December 28, 1881, aged 84 years.

DR. H. J. GARRIGUES has been appointed visiting physician to the Maternite Hospital, of New York, in place of Dr. W. T. Lusk, who has resigned.

THE MEDICAL SOCIETY OF VIRGINIA at its last meeting resolved to petition the Legislature of that State, to create a State Board of Medical Examiners.

AND STILL ANOTHER.—M. Gibier describes a new bacterium found in acute pemphigus. By the way, *is* there such a disease as "acute pemphigus"?

DR. OSCAR J. COSKERY, has been elected President of the Baltimore Medical and Surgical Society, for the ensuing year.

VACCINATION, according to Dr. J. L. Cilley, is generally performed in Cincinnati by barbers, who publish the fact on their signs that "Hier wird geimpft."

A CASE is reported in the *Lancet*, of maternity at nine years of age. The child-mother had menstruated from her first year, and was a full-formed woman at nine.

DR. JOSEPH F. EDWARDS has retired from the editorship of the *American Specialist*. The publication of the journal will be continued by Blakiston, Son & Co.

DR. T. B. CURTIS, of Boston, died in that city on the 11th of December. Dr. Curtis had just been appointed an instructor in Harvard University, as announced by us last month.

A CASE OF EXTREMELY SLOW PULSE is reported by Dr. J. Baranowitz in the *Medical Record*. The average number of beats per minute was about forty. The heart was normal.

M. PASTEUR, at the age of sixty, has gone upon a hunt after the microbe of yellow fever. Perhaps the Havana Commission of our National Board of Health could give him some points.

A DISPENSARY for the treatment of aural diseases has been established in connection with the Presbyterian Hospital of Philadelphia. Dr. C. H. Burnett will have charge of the service.

DR. T. LAUDER BRUNTON has contributed his endeavor to arrange the differences between religion and science by a work entitled "The Bible and Science." It has been very favorably received.

LISTERISM, according to Lawson Tait, "is one of the largest, best-blown, and most attractive bubbles ever displayed to a surgical audience." Mr. Tait will, before long, have a numerous following.

PREVIOUSLY TO SEWERING the city of Memphis, the death rate (exclusive of yellow-fever years) was thirty-five per 1000. During last autumn it was sixty per 1000. Is this a recommendation of the Waring system?

THE ROYAL ACADEMY of Belgium offers a prize of 1500 francs for the best essay on the effects of alcohol, physical and psychical, upon the individual and his offspring. The *concours* will close February 15, 1883.

DR. LOUIS ELSBERG has resigned his professorship in the Medical Department of the University of the city of New York, and has been elected professor of Laryngology and diseases of the throat, in Dartmouth Medical College.

PROF SSOR PIROGOFF, the distinguished Russian surgeon, is dead. His title to fame rests upon the amputation of the foot named after him, and upon an excellent atlas of topographical anatomy, illustrated by frozen sections of the body.

DR. KARL GROSSMAN, of Liverpool, and Dr. Priestly Smith, of Birmingham, are editors of a new monthly ophthalmological journal, which is published in this country by Blakiston, Son & Co., of Philadelphia. Its title is *The Ophthalmic Review.*

THE BOOK PUBLISHING firm of Lindsay & Blakiston has been dissolved by the retirement of Mr. Robert Lindsay. Mr. Presley Blakiston has associated with himself his son, and will continue the business at No. 1012 Walnut Street, Philadelphia.

AN INTERNATIONAL EXHIBITION of objects relating to public health and life-saving appliances will be held in Berlin, from June to October of this year. Mr. H. W. Fabian, of New York, is special commissioner for the United States. He will furnish all necessary information on application.

DR. S. H. WEEKS has accepted the Chair of Surgery in the Maine Medical School, made vacant by the death of Dr. William Warren Greene. Dr. Henry F. Gerrish, of Portland, has been appointed Lecturer on Anatomy, and Dr. Charles O. Hunt, of Portland, Lecturer on Materia Medica and Therapeutics.

A COMMISSION has been appointed by the Faculty of Medicine to report upon the study and practice of dentistry in France. They have decided to create a special dental diploma. Candidates for this will have to be twenty years old, must study dentistry two years in a school, and must have had one year's practical experience.

DR. JOHN W. DRAPER, formerly professor of physiology in the University of New York, and author of a "Treatise on Physiology," "Intellectual Development of Europe," "Conflict between Religion and Science," and other works of a high scientific and philosophical character, died on January 4th, in the seventy-fourth year of his age.

A NEW SOCIETY has been organized in New York city, called the Materia Medica Society. It has but two officers, who are chosen by lot, and not by ballot. Every person desiring to become a member must write an acceptable thesis. Its members now number thirteen, including some of the most eminent medical gentlemen in New York city.

M. BERGER advocates a method of skin-grafting, of exciting the vascularization of the flap before cutting it, by covering the skin either with a mustard plaster or with warm poultices. He has already found this method to be successful.

THE INOCULATIONS OF PASTEUR for splenic fever, are reported, up to October 1, on 160 flocks, containing 68,900 sheep. Before vaccination was practiced, the mortality from the fever was about one in twenty-three. During vaccination, and before its effects were fully obtained, about one in 130 died. After the operation had produced its full effects, there died only about one in 7,000 of those vaccinated.

A CONSULTATION seems to be a serious thing for the patients to judge from the following lines :—

A single doctor like a sculler plies ;
The patient lingers and by inches dies ;
But two physicians, like a pair of oars,
Waft him with swiftness to the Stygian shores.

IN A LETTER TO THE SAVANNAH, GA., *News*, Dr. W. G. Bulloch relates an incident in which a gentleman who was in the habit of dipping his face into a basin of hydrant water while bathing, suddenly felt drops of blood trickling from his nose, and after some trouble expelled a leech one inch and a quarter long from it. It is said on the authority of Dr. Parkes, that the French army in Algiers have suffered much from the men swallowing minute leeches, which caused dangerous internal bleeding.

